# What Makes a Foreign Language Intelligible? An Examination of the Impact of Musical Ability and Individual Differences on Language Perception and How Intelligible Foreign Languages Appear

**DOI:** 10.3390/jintelligence11030043

**Published:** 2023-02-23

**Authors:** Markus Christiner, Valdis Bernhofs, Sabine Sommer-Lolei, Christine Groß

**Affiliations:** 1 Centre for Systematic Musicology, Faculty of Arts and Humanities, University of Graz, Glacisstraße 27, 8010 Graz, Austria; 2 Jazeps Vitols Latvian Academy of Music, K. Barona Street 1, LV-1050 Riga, Latvia; 3 Austrian Academy of Sciences, Dr. Ignaz Seipel-Platz 2, 1010 Vienna, Austria

**Keywords:** intelligibility, singing ability, musical aptitude, short-term memory, perception, melody, difficulty, memorable, speech perception, language ability

## Abstract

Previous research suggests that musical ability is associated with language processing and foreign language pronunciation. Whether musical ability is associated with the ability to generate intelligible unfamiliar utterances has not been investigated. Furthermore, how unfamiliar languages are perceived has rarely been related to musical ability. We tested 80 healthy adults, with a mean age of 34.05 and a combination of 41 women and 39 men. We used batteries of perceptual and generational music and language measures to assess foreign language intelligibility and musical capacity. Regression analysis revealed that five measures explained the variance in the intelligibility of unfamiliar foreign utterances. These were short-term memory capacity, melodic singing ability, speech perception ability, and how melodic and memorable the utterances sounded to the participants. Correlational analyses revealed that musical aptitude measures are related to melodic perception and how memorable unfamiliar utterances sound, whereas singing aptitude is related to the perceived difficulty level of the language material. These findings provide novel evidence of the link between musical and speech abilities. In particular, intelligibility measures are associated with singing aptitude and how melodic languages appear to be. As impressions on how foreign languages are perceived are also related to musical capacities, perceptual language parameters address a new perspective that facilitates the understanding of the link between music and language in general.

## 1. Introduction

Previous studies provided evidence that musical and language abilities are linked and proposed a positive transfer from music-to-language (Christiner 2018; Ludke 2018; Ludke et al. 2014; Milovanov 2009; Milovanov and Tervaniemi 2011). This is not surprising since musical intelligence has been associated with several musical capacities such as perceiving, discriminating, performing, or expressing sounds (Gardner 1993), which are highly crucial in the early stages of foreign language learning where sound acquisition plays a dominant role. This is why linguistic and musical intelligence have been suggested to be intertwined (Zybert and Stępień 2009). Therefore, the question arises as to whether musical ability may also be a predictor of the ability to generate intelligible utterances of unfamiliar languages. Studies on overlapping elements of music and language have suggested that musical training mechanisms enhance the pitch and duration discrimination abilities of speech (Chobert et al. 2014; Moreno 2009). Positive relationships between both faculties have also been reported for the ability to segment speech (Christiner 2020; François et al. 2013), reading skills (Fonseca-Mora et al. 2015), phonemic awareness (Gromko 2005), pronunciation skills (Christiner and Reiterer 2015), and verbal memory (Ho et al. 2003; Moreno et al. 2011), among many others. In particular, singers and instrumentalists performed better in language measures that focused on generational processes such as pronunciation tasks (Christiner and Reiterer 2015). Even though we also noted that musicians were better at speech perception, the difference between musicians and non-musicians was more dominant and consistent in language abilities, focussing on imitation and pronunciation skills (Christiner 2020).

Other researchers determined that the relationship between musical training and language capacity may be overestimated (Swaminathan and Schellenberg 2020). These studies suggest that the relationship between musical and language ability is less influenced by training mechanisms and arises primarily from preexisting factors (Kragness et al. 2021; Swaminathan and Schellenberg 2020). This is in line with aptitude research, which suggests that training effects cannot fully account for explaining individual differences in musical (Butkovic et al. 2015; Mosing et al. 2014; Pulli et al. 2008) and language abilities (Golestani et al. 2011). This is also plausible since it is a commonly accepted notion that individuals with the same amount of training in music or language do not necessarily reach an equal level of proficiency. Therefore, innate, genetic, or early acquired factors have been brought into play in language and music aptitude research (Christiner and Reiterer 2018). These factors should explain the individual differences in musical (Pulli et al. 2008; Seither-Preisler et al. 2014) and language ability (Golestani et al. 2011), which cannot be explained by training effects.

Singing is another musical capacity that has been related to language ability (Christiner and Reiterer 2015; Christiner et al. 2022b; Ludke 2018; Ludke et al. 2014). Singing songs can be seen as a kind of hybrid category consisting of language and musical components (Christiner and Reiterer 2019). Singing as a tool facilitates the memorization of new vocabulary, which is improved if utterances are sung (Ludke et al. 2014). The singing ability has also been a good predictor for explaining individual differences in the ability to acquire the foreign language pronunciation of typologically different languages (Christiner 2020; Christiner et al. 2022c; Ludke et al. 2014). More recently, it has been shown that individuals who sang more frequently during childhood also had better foreign language pronunciation skills during adulthood (Christiner et al. 2022c). The singing benefit has been associated with two aspects. One is enhanced vocal-motor skills and sensorimotor ability, which explains why individuals who sing well also pronounce new languages well (Christiner et al. 2022a, 2022c). The second is the singing component melody, which may function as a mnemonic or a memory booster (Christiner et al. 2021, 2022a, 2022c). Melody is not only an element that facilitates the memorization of songs but also helps the learning of new words (Thiessen and Saffran 2009) and the recollection of song lyrics (Purnell-Webb and Speelman 2008). Melody serves as a kind of mnemonic with which utterances are probably stored in long-term memory (Gordon et al. 2010). Individuals with higher musical aptitude and expertise can incorporate new sounds of languages more easily (Kraus and Chandrasekaran 2010), remember longer sound chunks (Pastuszek-Lipinska 2008), and possess enhanced short-term memory (STM) capacity (Christiner and Reiterer 2013).

The role of STM has been discussed in the context of both music (Christiner and Reiterer 2013; Christiner et al. 2022b; Coumel et al. 2019) and (foreign) language research (Dörnyei 2005; Dörnyei and Ryan 2015; Wen et al. 2017; Wen and Skehan 2011). Evidence for the crucial role of STM in language learning comes from multiple sources. For instance, impairment studies suggest that poor foreign language performance stems from deficits in the phonological loop (Baddeley 2010; Baddeley et al. 1998). Approaching from the opposite direction, exceptional learners of foreign languages possess improved phonological STM capacity (Biedroń and Pawlak 2016). STM capacity is one of the most dominant predictors explaining individual differences in language performance, particularly in initial language learning situations (Christiner 2020; Gathercole and Baddeley 1990; Payne and Whitney 2002). Recent research suggests that the processing of musical and verbal sounds corresponds only slightly in the auditory STM (Williamson et al. 2010), even though the storage of verbal and tonal signals seems to rely on overlapping neuronal networks and may not be entirely separable (Koelsch et al. 2009). Since STM capacity plays a crucial role in language and, more recently, has also received more attention in music research, STM measures should be part of studies that focus on music and/or language performance.

Research has also noted that whether something is perceived as language or music, it can be easily manipulated. This so-called speech-to-song illusion has shown that spoken language can be transformed to sound like the song when the same language stimuli have been repeated several times (Deutsch et al. 2011). The speech-to-song illusion also occurred more often when the language pronunciation tasks appeared to be more difficult (Margulis et al. 2015). A similar observation has also been provided more recently. The melodicity of languages appeared to predict how well they were pronounced (Christiner et al. 2021). Therefore, perceptual parameters for how foreign languages are perceived may be a promising new research field that should be integrated into research on the overlapping elements of language and music.

In previous research, we examined the relationship between pronunciation skills and various music variables (Christiner et al. 2021, 2022a, 2022b). However, whereas previous studies assessed pronunciation skills holistically with no particular focus on specific language features, we selected to focus on the intelligibility of newly produced unfamiliar languages and its relationship to musical ability, STM ability, and perceptual language variables in this study.

**Musical measures**

Various measurement protocols have been developed that aim to measure individual musical skills and provide information about musical aptitude, musical experience, and sophistication. Most musical and elementary ability tests are perceptual measures that are based on similar conditions. These tests primarily focus on rhythmic or tonal discrimination tasks (Gordon 1979; Gordon 1982; Gordon 1989a, 1989b; Law and Zentner 2012; Wallentin et al. 2010). Complex music measures as used in Gordon’s Advanced Measures of Musical Audiation (AMMA) have also been criticized. It has been suggested that they may measure a combination of skills (Law 2012; Law and Zentner 2012). More recently developed aptitude measures such as the Profile of Music Perception Skills (PROMS) focus on less complex stimuli than Gordon’s tests and aim to be more culturally neutral (Law and Zentner 2012).

While there are large numbers of perceptual measures, musical performance tasks are less often used to assess musical ability. At the same time, there is a considerably smaller amount of musical performance measures. The latter include hand-claps, memorizing music phrases, sight reading, improvisation tasks, repeating melodies, or singing (Christiner et al. 2022a, 2022b; Groß et al. 2022; Wallentin et al. 2010). This may be related to the fact that musical performance measurements are difficult to analyze as they are often rated and evaluated by experts. Since many studies reported contradictory results when the link between music perception and performance was assessed (Berkowska and Dalla Bella 2009), the inclusion of music perception and performance measures will give a more detailed basis for individual differences in musical capacities. Therefore, we included measures of musical aptitude that focus on musical perception as well as singing tasks as musical performance measures. The latter are measurements that can also be targeted at non-musicians.

**Language measures**

Most standardized language ability measures focus on novel and artificial language learning processes, the application of grammar rules, recognizing visual cues and sound relationships, and/or individual differences in perception and memorization (Carroll 1958; Carroll and Sapon 1959; Meara 2005; Parry and Child 1990). As we have seen for musical ability measures, research on language ability often focuses on perceptual measures, although research indicates that accurate perception does not always predict accurate pronunciation (Golestani and Pallier 2007). Therefore, a well-rounded research design should also include performance measures to test language skills, aside from criteria for perceptual language assessment.

However, there appears to be no standardized language measure available that focuses on assessing language performance, such as foreign language pronunciation or imitation. One reason may be that the evaluation of pronunciation tests requires a greater investment in the study since they must either be analyzed acoustically or rated by native speakers or language experts (Christiner 2020). However, pronunciation tasks have high ecological validity as they simulate the natural learning setting in which new languages are acquired. The assessment of the nature of pronunciation tasks in various foreign languages revealed that short sequences of unfamiliar utterances between nine and eleven syllables provide individual differences in pronunciation skills in more general terms (Christiner 2020). This means that the typological differences of the languages play a lesser role in pronunciation tasks if they do not focus on specific language features. As a result, different language pronunciation tasks—which we also use in the current study—represent a more general pronunciation aptitude measurement and can be used as a single test (Christiner 2020).

Studies assessing pronunciation or imitation ability show many similarities to research that focuses on individual differences that predict the intelligibility of foreign-accented speech. They both focus on assessing foreign language pronunciation performance, which has to be rated by another group of participants (Gooskens 2013). However, while in language aptitude research it is often common to use language stimuli unfamiliar to participants (Meara 2005), research on language intelligibility focuses on selecting participants who have already acquired knowledge in the foreign languages that are being tested. Therefore, more common approaches to assessing intelligibility make use of accented foreign speech (Bent and Bradlow 2003; Munro and Derwing 1999; Pérez-Ramón et al. 2022; Pinet et al. 2011).

**Aims and hypotheses**

In this study, we looked at the intelligibility of foreign languages from a different approach, and we selected language material that is commonly used to assess language pronunciation aptitude. Therefore, we used our previously developed language measures, which were completely unintelligible to the participants, to assess individual differences in the ability to reproduce intelligible utterances. While factors influencing the degree of intelligibility are often studied from a sociolinguistic or dialectical perspective, our goal was to assess the relationship between being able to generate intelligible sounding unfamiliar languages and musical abilities in more detail for several reasons. First, enhanced musical abilities and phonetic skills require being sensitive to acoustic information (Christiner et al. 2021, 2022a, 2022b), and second, the degree of intelligibility of sung lyrics have also been associated with the vocal ability (Novák and Vokrál 2000). Since both vocalizations require the integration of acoustic and sensory-motor information, our first hypothesis was that singing ability would be associated with the ability to generate intelligible speech in unfamiliar languages (H1). In previous research, we also noted that the melodic perception of utterances was associated with the ability to imitate unfamiliar languages and musical aptitude (Christiner et al. 2021). Therefore, besides the melodic perception criterion, we introduced further behavioral language rating criteria where the participants had to indicate how pleasant-sounding, memorable, familiar, and difficult to mimic the language samples appeared to be. We suggested a positive association between the behavioral language rating criteria and the ability to generate intelligible unfamiliar utterances, except for familiarity (H2). The latter criterion was included as a further control variable and was expected not to be associated with the intelligibility measure since the participants did not speak or comprehend one of the selected foreign languages. We also expected to find positive associations between musical ability measures and the behavioral language rating criteria, melodic perception, pleasant-sounding, memorable, and difficult to mimic (H3).

## 2. Materials and Methods

### 2.1. Participants and Raters

The study consists of two different participant samples. The first represents participants (N = 80) who were tested for language, music, and STM ability, and the second represents raters who assessed the language imitation tasks of the respective six different languages (N = 27) and the two singing tasks (N = 4).

#### 2.1.1. Participants

The study was advertised on notice boards and social media platforms. The recruitment criteria for participation included the following conditions: They should (1) be German native speakers who received instructions in foreign languages when they entered school; (2) not be able to speak or comprehend one of the languages which were selected for this research; (3) have at least secondary academic school as the highest level of education; (4) be full-time students during the testing time or should have successfully finished a course of study previously; (5) participate voluntarily in the experiment. In addition, the participants reported that they did not possess cognitive, neurological, or hearing impairments. Furthermore, the participant’s elementary hearing ability was also assessed by using the KLAWA (Klangwahrnehmung) measure as used in previous investigations (Christiner et al. 2022b; Schneider et al. 2022). The results indicated that all participants were within the expected normal hearing range. The mean age of the participants was *M* = 34.05, *SD* = 11.18. There were 41 female participants and 39 male participants. We also assessed whether we could detect gender differences in the ability to generate intelligible utterances by performing a *t*-test. Results indicated that the intelligibility measures did not significantly differ between male (*M* = 2.4, *SE* = 0.15) and female (*M* = 2.6, *SE* = 0.17) participants *t*(78) = −1.06, *p* = 0.29. Thirty-five participants indicated that their highest level of education is a secondary academic school; eighteen possess a bachelor’s degree, while twenty-seven have a master’s degree. The Ethics Committee of the Institutional Review Board of the Medical Association of Riga, Latvia (2-PĒK-4/3/2022), study’s procedures. The participants took part in the study voluntarily.

#### 2.1.2. Raters

We recruited raters for each of the respective six languages who assessed the intelligibility of the participants’ performances. The rater had to be an adult native speaker with a linguistic background. The mean age was *M* = 35.85, *SD* = 6.43. The rating sample consisted of 17 female and 10 male raters. They all had either a master’s or doctoral degree and received compensation for their rating.

The singing raters were all professional singing teachers who had either a bachelor’s or master’s degree. The mean age was *M* = 45, *SD* = 3.63, and two of the singing raters were male and two were female. They also received compensation for their work.

### 2.2. Language Intelligibility

The language intelligibility measurement consisted of language samples in Thai, Mandarin, Tagalog, Farsi, Japanese, and Russian. These languages were completely unfamiliar to the participants. The language samples of each of the selected languages had either nine or eleven syllables. The language stimuli spoken by the native speakers were of moderate speed and resembled spoken natural language. The original language files were recorded in a sound-proof room with the music software Steinberg CUBASE 8.

The participants had to repeat four phrases from each of the six languages. We had four different speakers (two male and two female) for each of the six languages. Before testing took place, a familiarization task was provided. The participants listened to four Turkish and Slovak language samples. Each sample was separated by a pause of 50 ms and played three times before the participants repeated them. After the participants performed the familiarization task, testing of the respective six languages took place. The participants wore headphones with an integrated microphone (Beyerdynamic DT 290) while they listened to the original sound files and while their performances were recorded. The recordings of the participants were also performed with the music software Steinberg CUBASE 8 to have high-quality samples. The sound files were normalized for loudness by a sound technician.

#### Language Raters and Rating Procedure

The language performances were rated on an online rating platform by the native speakers and linguists of the respective languages. The raters were instructed to listen to both the original sentences spoken by the native speaker of the respective language and the performances of the participants. The raters were instructed to evaluate how intelligible the language samples appeared to them. The rating scale ranged from 0 “min” to 10 “max”, where 0 meant that the samples were completely unintelligible and 10 meant they were completely intelligible and native-like. For assessing interrater reliability, intra-class correlation coefficients were performed. We had three raters for Russian, six for Japanese, four for Tagalog, five for Mandarin, four for Farsi, and five for Thai. The ratings indicated that all ratings were equal to or above the accepted value of 0.7. Detailed information about intra-class correlations is contained in the supplement (see Tables S1–S6). All raters received compensation for their work. We calculated a total score that consisted of all language samples (intelligibility).

### 2.3. Behavioral Perceptual Language Ratings

We used the research design of previous research on the melodic perception of unfamiliar languages (Christiner et al. 2021). In the current study, we added new behavioral language ratings that focus on the participants’ impression of how pleasant-sounding (pleasant-sounding), melodic (melodic perception), difficult to mimic (difficulty level), memorable (perceived memorability), and familiar (familiar) the language samples appeared to them.

The first step of testing included a participant familiarization stage to introduce them to the meaning of five behavioral ratings. By memorable, we introduced the participants to indicate how memorable the language material appeared to them. By pleasant-sounding, we explained that they should indicate the degree of listening enjoyment of the language samples. We then explained that with melodic, the participants should indicate how musical and melodic the language samples sounded to them. The criterion of difficulty level meant that the participants were instructed to estimate the difficulty level to mimic the language samples, while familiarity referred to whether the language material sounded familiar to them. The criterion of familiarity was introduced as a further control variable as the language material was completely unfamiliar to the participants.

After the participants were introduced to the rating criteria, they had to listen to the same original foreign language samples for the intelligibility measure again. They listened to the four samples of each language in a row and had to indicate their response afterward. They began by familiarizing themselves with Turkish and Slovak. After they indicated that they had understood the task, testing of the six languages—Thai, Mandarin, Tagalog, Farsi, Japanese, and Russian—took place. The ordering of the behavioral language judgments was randomized. For instance, the first round could include a participant being instructed to indicate how memorable the language samples appeared to her/him on a scale between zero and ten. The language sample score of ten was the highest value (very memorable), and zero was the lowest value (not memorable). For the testing procedure, the participant listened to four different language samples of the same language (e.g., Thai) in a row. Then, she/he had to indicate her/his judgment about how memorable the four samples of the respective language appeared to be. Subsequently, the next four samples of one of the other languages followed. The participants did not receive any information about which language they were rating, which aims to reduce rating differences that may be influenced by positive or negative associations and attitudes toward the selection of our languages. After the perceived memorability was rated, the participants had a 1-min break before one of the next four criteria (melodic perception, pleasant-sounding, difficulty level, and familiarity) was rated. As for the intelligibility measurement (see Section 2.2), we treat the individual languages as a single test design, which means that we calculated total scores comprised of the ratings of all six languages for each of the five criteria. We named them pleasant-sounding, melodic perception, difficulty level, perceived memorability, and familiarity.

### 2.4. Language Perception Aptitude

While for the intelligibility measurement (see Section 2.2) and the behavioral language ratings (see Section 2.3), the same language stimuli were used, the language perception measurement is independent of the former measures. It assesses individual differences in the ability to discriminate and remember unintelligible language stimuli. We used the subtests in Thai, Tagalog, Mandarin, Farsi, and Japanese since these five languages were not spoken or comprehended by any of the participants. A detailed description of the test design can also be found in Christiner (2020) and Christiner et al. (2022c).

The language measurement consists of a familiarization task and a main measurement of twenty-five tasks (five for each language). The test design consists of language strings (Stimline), which can be comprised of eight, ten, or twelve different words or short phrases (Stims). After the participants have listened to the entire Stimline, a comparative string (Stimcompare) follows. The Stimcompare can also be composed of one, two, or three words or short phrases, depending on the difficulty level. The Stims of the Stimline are separated from each other by a pause of 50 ms, whereas the Stimcompare is separated from the string by a pause of 2 s. A change in the color of the screen also indicates the transition from Stimline to Stimcompare. The Stims were always spoken by the same speaker, which is why each task sounded like a sequence of foreign speech.

The participants were instructed to indicate whether the Stimcompare was included in the Stimline they were listening to before or not. If the Stimcompare was included in the Stimline they had to click the correct button. If the Stimcompare was not contained in the Stimline they had to click the incorrect button. Participants were instructed to click on the correct button only if all comparative phrases were included in the string when the Stimcompare consisted of more than one Stim (two or three). The sum of all twenty-five items represented the language perception aptitude score. Participants received one point for each correct answer. The values of the language perception aptitude score are presented as percentages of correct answers in decimal numbers (see descriptive Table 1).

### 2.5. Short-Term Memory

For assessing individual differences in STM capacity, we used the Wechsler Digit Span (Wechsler 1939). This digit span is well-known and consists of a forward digit span subtest and a backward digit span subtest. The digit sequences vary between three and nine digits in the forward subtest, while the backward span subtest is comprised of sequences between two and eight digits. The test was programmed online, the stimulus was presented acoustically, and the responses of the participants were automatically scored. The participants were instructed to repeat the steadily increasing sequence of digits in either a forward or a backward order. We calculated separate scores, one for the forward span (short-term memory forward) and another for the backward span (short-term memory backward). The maximum score that can be reached is fourteen for each of the two subtests.

### 2.6. Musical Aptitude: Advanced Measures of Music Audiation (AMMA)

The AMMA test, developed by Gordon (1989a), has been used to assess musical aptitude in multiple investigations and provides information about individual differences in the ability to discriminate rhythmic and tonal changes in paired musical statements. This musical aptitude measure focuses on tonal and rhythmic discrimination abilities. The paired musical statements are embedded in a single test design in which either rhythmic, tonal, or no changes can occur. When the melody is played for the second time in the tonal subtest, the notes are modified in a different condition, whereas in the rhythm subtest, the duration, tempo, or meter are modified in a different condition. The AMMA test is used to assess individual differences in musical ability in adolescents and adults. The test consists of a familiarization section, which consists of three items. After participants are introduced to the test, thirty items follow. The test generates three scores, which are based on an algorithm that was developed by Gordon (1989a). The tonal and rhythmical scores, as well as the total score (which includes the tonal and rhythmic subtests), are generated. The scores for the tonal and rhythmic subtests range between zero and forty. Since the total score is only the sum of the two subtests, we only took the tonal (AMMA tonal aptitude) and rhythmic (AMMA rhythmic aptitude) subtests for further analysis.

### 2.7. Singing Aptitude

For assessing individual differences in the ability to sing, we used parts of the previous singing test design in which two parts of an unfamiliar song had to be learned. We used the two short imitation tasks where we knew that these two tasks were managed by both musicians and non-musicians (Christiner 2020; Christiner et al. 2021). The two parts of the song belong to the opening of an unfamiliar song. Part one is the shorter sequence of the two singing tasks. The second singing task is an extension of the first and therefore considerably longer. The lyrics of part one are “whenever I miss, whenever I miss, I miss your smiling”, while for the second part, the lyrics were “whenever I miss, whenever I miss, I miss your smiling, whenever I try, I try to fake a little smile”. With this measure, we aimed at assessing how fast and accurately participants were able to repeat and learn a new unfamiliar song. This way of assessing singing ability is comparable to music and language aptitude measures.

The singing task was divided into two different parts, which became increasingly difficult. The participants had to listen to the original part of the song three times before they had to sing that part of the song. The participants had to repeat and sing the part of the song purely from their memory without background music. In addition, the participants were also allowed to sing the part of the song in a key that suited their personal singing voices, as the key was not an assessment criterion for the singing voices. The short sequences of the song are provided in the supplement (Figure S1).

#### Raters and Rating Procedure

For assessing individual differences in singing aptitude, we followed procedures from previous research (Christiner and Reiterer 2013; Christiner et al. 2018, 2022a). The recordings of the participants’ singing performances were rated by four singing experts for how consistently and well the participants had sung the melody (melodic singing aptitude) as well as how accurately the participants were able to sustain the original rhythm (rhythmic singing aptitude) of the song. The rating scale ranged from zero to ten, with zero being the lowest score and ten being the highest. We decided to use these rating criteria since they represent an equivalent to the musical aptitude test AMMA, which consists of a tonal and rhythmic subtest. For assessing the interrater reliability of the ratings, intra-class correlation coefficients were performed. The results indicated that the ratings for melody and rhythm were above the accepted value of 0.7. Detailed information about the values is contained in the supplement (see Table S7).

### 2.8. Testing Process

The testing of participants was divided into different steps. First, the participants were instructed to provide background information on an online platform, with which we were able to pre-select our participants according to our recruitment criteria. Then the participants were invited to a lab two times for about seventy minutes each. In the first session, we verified the background information that was initially provided online. Afterward, the participants were assessed for their elementary hearing ability, followed by the musical and, finally, the language perception measures. In the second session, the participants first performed the language pronunciation tasks with which we measured intelligibility. Next, they listened to the language samples from the intelligibility task again and performed the behavioral language ratings. Finally, the STM task was performed. Although testing took place mainly in the lab, the AMMA test, the STM, and the language perception measures were performed online. This should equalize testing conditions and make data collection more resistant to errors.

The raters who assessed the language and singing performances rated the performances online. Each rater was instructed on the rating criteria in person, and the rating criteria were additionally described on the online platform.

### 2.9. Statistical Analyses

The statistical analysis is divided into four main sections. First, we provide the descriptions of the variables (see Table 1). Second, we performed correlational analyses for all variables to outline their relationship. Third, after a close inspection of the correlations’ matrix, we performed a stepwise multiple regression where the intelligibility score was the dependent variable. With this analysis, we wanted to provide information about the predictors involved in predicting the intelligibility of language performances. Fourth, we also performed a correlational analysis for the behavioral language ratings and the musical variables to uncover whether the specific criteria of the behavioral perception of languages are also related to musical abilities.

## 3. Results

### 3.1. Descriptives

Table 1 below illustrates the descriptives characteristics of the variables under consideration.

### 3.2. Correlational Analyses

We performed correlational analyses to uncover whether the intelligibility score was related to the variables under consideration. Table 2 shows the correlations between the intelligibility score and the musical variables under consideration, while Table 3 illustrates the table of the correlations between the behavioral language ratings and the STM measures under consideration.

### 3.3. Regression Analysis

We also performed multiple linear regressions. In the regression model, the variables that correlated with the intelligibility score were entered into a multiple linear regression as independent variables. The independent variables were included in the multiple linear regression models only if a probability of F-change < 0.05 was given. We decided to use a stepwise method. As there is a lack of theoretical foundation for the predictors and language intelligibility, the stepwise regression model is one way to search for patterns in the dataset that is based on purely mathematical decisions. In addition, the stepwise model was used to reduce the number of variables. We entered all music and language variables simultaneously. The results revealed that fifty-four percent of the variance in the intelligibility score could be explained by five predictors. These are the STM forward: melodic perception, melodic singing aptitude, language perception aptitude, and perceived memorability (see Table 4).

### 3.4. Correlational Analyses of the Behavioral Perceptual Language Ratings and the Musical Variables

We performed correlational analyses to uncover whether the behavioral language ratings were also related to the musical variables. Table 5 show the correlations between the behavioral ratings, melodic perception, perceived memorability, difficulty level, and the musical variables under consideration. Since the behavioral language ratings of familiarity and pleasant-sounding did not show any correlations to musical variables, we included them for transparency reasons only in the supplement (see Tables S8 and S9).

## 4. Discussion

In this investigation, we addressed three hypotheses. The first focuses on the relationship between being able to generate intelligible foreign utterances and previously used language, STM, and musical measures (H1), and the second focuses on the participants’ impressions of how pleasant-sounding, melodic, difficult-to-mimic, memorable, and familiar the unfamiliar foreign languages sound (H2). To find the most important variables for explaining individual differences in the intelligibility score, we performed a regression analysis in which all language, music, and STM variables that correlated with the intelligibility score were entered (see Section 3.2 for the correlations and Section 3.3 for the regression model). The findings revealed that the variance in the degree of intelligibility could be explained by five measures, namely STM forward, melodic perception, melodic singing aptitude, language perception aptitude, and how memorable the language samples appeared to the participants. Our first hypothesis has been confirmed, and the singing aptitude criterion (melodic singing aptitude) remained the only musical measure that was associated with the intelligibility performance. Parts of our second hypothesis have also been confirmed. The behavioral language rating criteria of melodic perception and perceived memorability partly explained the variability in being able to generate intelligible utterances, while as expected, familiarity was not associated with the dependent variable. Although the variable difficulty to mimic was correlated with the measure of intelligibility, it did not reach significance in the regression model, and pleasant sounding was not related to the degree of intelligibility at all.

In light of the present findings, we divided the discussion on hypotheses H1 and H2 into two sections. The first section discusses variables that represent previously used measures. These are STM, language perception, and singing aptitude. In the second section, we provide possible explanations for why behavioral language measures such as melodic perception and perceived memorability contribute to the ability to generate intelligible foreign utterances.

**Short-term memory, language, and singing aptitude**

One of the most important predictors of individual differences in language ability is short-term memory ability (Christiner and Reiterer 2018; Dörnyei 2005; Hummel 2009; Robinson 2002, 2005; Wen et al. 2019). In this investigation, the forward and backward span correlated with the intelligibility score, but the forward span was only associated with the intelligibility measure in the regression model. The forward span resembles non-word repetition measures and the learning of short sequences of new languages, while the backward span focuses more on controlled attention (Engle et al. 1999). This could be why the forward span contributes more likely to language performance tasks, as previously observed in research (Christiner 2020; Christiner et al. 2018).

The phonological STM capacity was measured by using a digit span (forward and backward) in which the participants had to repeat a steadily increasing sequence of digits. This capacity is rather crucial for the acquisition of new languages. Individuals that retain a larger number of phonetic elements in a short period of time are also able to incorporate new sounds into a language-learning situation more easily (Hummel 2009). In the early stages of (foreign) language learning, the phonological loop capacity is a crucial element that is associated with foreign language success. The intelligibility measure of our study was designed to simulate an initial foreign language learning situation, which is why we suggested that improved STM capacity would be associated with being able to generate intelligible utterances.

Another measure that was able to explain individual differences in the degree of intelligibility was the language perception measurement. This language measurement assesses individual differences in the ability to perceive unfamiliar language. In general, it can be argued that perception precedes language material reproduction, and thus a relationship between speech perception ability and how intelligible newly learned utterances sound was expected. However, the opposite was also found in previous studies. Individuals who perceived foreign speech sounds more accurately did not necessarily pronounce them better (Golestani and Pallier 2007). In another study, mixed results between language perception and language pronunciation tasks were observed. Two out of four languages did not show associations between language pronunciation and perception tasks (Christiner 2020). However, in the previous study, fewer language samples were used. Given the results of this study, it can be suggested that enhanced perceptual skills and the ability to reproduce intelligible utterances are interrelated.

We also expected that the degree of intelligibility of the language performances would be associated with singing aptitude. The participants had to learn an unfamiliar song as accurately as possible in a short time. Previous research has provided evidence that singing ability is related to language abilities that focus on mimicry, imitation, and pronunciation ability (Christiner and Reiterer 2013; Christiner et al. 2018, 2022b; Coumel et al. 2019). In previous research, the singing benefit was related to enhanced vocal-motor skills and sensorimotor ability, which should facilitate both singing and language pronunciation skills (Christiner et al. 2022a, 2022c). In light of the present findings, it can be suggested that improved singing ability makes newly learned utterances sound more intelligible.

As a result, in the pedagogical context, incorporating singing as a tool to improve foreign language intelligibility may be a promising way to improve pronunciation skills. For instance, this could include exercises in which words or phrases in a foreign language are sung. However, a more promising way would be to teach foreign languages and musical abilities simultaneously or within the same framework. This would probably improve both foreign language and musical abilities.

The melodic component of singing seems to play a particular role. Research on adults and children has shown that the learning of new vocabulary improves if the new vocabulary is sung and learned together with a melody (Ludke et al. 2014; Thiessen and Saffran 2009). Vowel intelligibility decreases with increasing pitch height in singing (Sundberg 1977). As individuals with the better singing ability also have a larger vocal range (Sundberg 1988), it can be suggested that singing ability increases the ability to pronounce intelligible utterances. However, singing is not always considered a beneficial tool for acquiring new languages. This is the case when specific language features are learned (Christiner et al. 2022b). For instance, the learning of Mandarin syllable tones requires high tonal precision, which is altered and neutralized when Mandarin tone syllables are sung (Christiner et al. 2022b).

Although the tonal and rhythmic musical aptitude measures were correlated with the intelligibility scores, they did not turn out to predict individual differences in the language pronunciation task in the regression model. This finding has been replicated several times in behavioral research (Christiner 2020; Christiner and Reiterer 2013; Christiner and Reiterer 2019). One explanation for the closer relationship between singing and language pronunciation tasks may be that both involve the ability to self-monitor and integrate sensory and vocal tract-related motor representations (Stager et al. 2003). Neurophysiological testing supports this hypothesis and has shown that enhanced singing and language pronunciation skills were both correlated with reduced N1 latency (Christiner et al. 2022a). This reflects, in addition to (pre)attentional processing, sensory stimuli processing, and sensorimotor integration (Giard et al. 1994; Näätänen and Picton 1987; Sharma et al. 1997).

**Intelligibility and behavioral language ratings**

The regression model revealed that the individual differences in the intelligibility measure could also partly be explained by two behavioral language rating predictors, namely the perceived memorability and the melodic perception of the language stimuli. The predictor of memorability represents a self-perception concept that reflects beliefs about the state of one’s abilities. This refers to how well the participants claim to be able to memorize the languages. The role of self-perception and language proficiency has been investigated in educational research in much detail. Research has found that self-perception is positively related to language performance (Dermitzaki and Efklides 2000; Onwuegbuzie et al. 1999). Similar findings were also observed in the current study. The perceived memorability of the languages was associated with the degree of intelligibility of the foreign languages. The more memorable the language material appeared to the participants, the more intelligible the utterances sounded to the native speakers of the respective languages.

The second behavioral predictor, melodic perception, was already found to explain individual differences in language pronunciation skills in previous studies. Individuals who perceived languages as more melodic also possessed more elaborate pronunciation skills in foreign languages (Christiner et al. 2021). Melody plays a key role in music and language memorization processes. New vocabulary is more easily remembered and retained if it is presented together with a melody (Ludke et al. 2014; Thiessen and Saffran 2009). The impression of melodic aspects contained in the language is associated with how intelligible newly learned utterances sound. This could also be related to the language stimuli we used. The languages were meaningless to the participants. In initial learning settings where the language material is poor in linguistic content, the acoustic features play a more important role. Since music and language consist of a set of similar properties such as pitch, timbre, and timing (Kraus and Chandrasekaran 2010), it could be suggested that in a situation in which individuals learn a new language, similar cognitive mechanisms may be activated like in listening to music.

This notion becomes evident when considering a phenomenon that seems to activate analogous cognitive mechanisms in both language and music. The din, or involuntary mental rehearsal ability, refers to a phenomenon in which acoustic information, in particular, melodic information, reoccurs without the initial effort of the speaker (Salcedo 2010). This is similar to musical or new foreign language input. The cognitive mechanism known as “musical din” (Murphey 1990) has a less well-known equivalent described in language. The “language din” is described as a process in which newly heard or learned utterances repeat without the speaker’s intentional effort (Salcedo 2010). The “language din” is more common in beginning language learners (Krashen 1983; Salcedo 2010) and is thought to be caused by the stimulation of a language acquisition device (Murphey 1990). The stimulation is best achieved with aural and unfamiliar input. Therefore, the musical and the language din seem to be activated by similar devices, namely, by acoustic, probably melodic, and new information.

**Musical abilities and behavioral language ratings**

Our third hypothesis was that we suggested finding positive associations between musical ability measures and the behavioral language rating criteria, melodic perception, pleasant-sounding, memorable, and difficult to mimic (H3). Therefore, we run a correlational analysis between the behavioral language ratings and musical variables (see Section 3.4). The findings revealed that melodic perception was related to tonal and rhythmic musical aptitude, perceived memorability was related to rhythmical musical aptitude, and perceived difficulty level was related to melodic and rhythmic singing aptitude (see Table 5. Pleasant-sounding and familiarity did not correlate to any of the musicality variables (see Tables S8 and S9 in the supplement). The correlation between the melodic perception of languages and musical aptitude has already been found in previous research and suggests that musical aptitude may be related to whether a language is perceived to be more melodic than others (Christiner et al. 2021). The correlation between perceived memorability and rhythmic musical aptitude is rather low and should not be overstated. Research has shown that individuals with higher musical aptitude and expertise were able to incorporate new sounds of languages more easily (Kraus and Chandrasekaran 2010) and could also remember longer sound chunks (Pastuszek-Lipinska 2008). It has also been suggested that the relationship between musical training and language may be mediated by memory capacity (Fennell et al. 2021). Therefore, future research could focus on the impression of how memorable languages appear and their relationship to musical variables in more detail.

Interestingly, the perceived difficulty level of the languages was correlated with both singing measures. Psychological research has shown that spoken language can be transformed into a sound such as a song (Deutsch et al. 2011). This phenomenon referred to as the speech-to-song illusion phenomenon, is achieved by repetition of the same language stimuli. A study that used the speech-to-song illusion paradigm proposed that the speech-to-song illusion occurred more readily when the speech material was more difficult to pronounce (Margulis et al. 2015). The positive relationship between rhythmic and melodic singing ability suggests that the better the singing, the more the participants were aware of the difficulty level of the language material. This may show that individuals with higher vocal skills are also better at estimating difficulty levels of vocalization in general. This would also be in line with self-estimation measures about singing skills, which are highly interrelated to participants’ singing performance and the ability to imitate foreign accents (Christiner 2020; Coumel et al. 2019).

The study has limitations. We only used a digit span (STM task) to assess participants and self-report questions to determine whether they had cognitive impairments. We did not include further measures of general cognitive abilities since this would have required the inclusion of further tests and increased testing time. Therefore, future research of a similar kind should also include a battery of measures of general cognitive ability.

Future research should focus on how perceptual ratings of language impressions relate to musical capacity in more detail since this approach could be a promising research field. It could contribute to explaining overlapping elements of language and music from a less well-understood perspective. Additionally, it might also be worth looking at listeners of tonal languages and non-tonal languages and whether the melodic perception of languages has differential implications for their speech intelligibility in congruent (other tonal/non-tonal languages) and incongruent (other non-tonal/tonal languages) languages.

## 5. Conclusions

Our findings show that the intelligibility of newly learned utterances is affected by STM ability, language perception ability, singing ability, and participants’ impressions of how melodic and memorable the languages appeared. Singing aptitude was the only musical ability measurement that was also associated with being able to generate intelligible utterances in the regression model, showing their close relationship. In addition, we also provided evidence that perceptual parameters, which describe how utterances are perceived, not only contribute to explaining the degree of intelligibility of newly learned utterances but are also related to musical capacities. This suggests that listening to unfamiliar languages may activate analogous cognitive mechanisms, such as listening to music.

## Figures and Tables

**Table 1:** Descriptives.

Variables	Mean (*M*)	Standard Error (*SE*)
Intelligibility	2.52	0.11
AMMA tonal aptitude	26.66	0.59
AMMA rhythmic aptitude	29.26	0.48
Melodic singing aptitude	5.97	0.18
Rhythmic singing aptitude	6.97	0.15
Short term memory forward	7.50	0.18
Short term memory backward	7.39	0.20
Language perception aptitude	0.65	0.02
Pleasant-sounding	5.53	0.16
Melodic perception	5.87	0.14
Difficulty level	6.88	0.19
Perceived memorability	4.75	0.15
Familiar	4.64	0.20

**Table 2:** The correlations between the intelligibility measure and the musical variables.

Variable	AMMA Tonal Aptitude	AMMA Rhythmic Aptitude	Melodic Singing Aptitude	Rhythmic Singing Aptitude
Intelligibility	.414 **	.334 **	.349 **	.365 **

** *p* < .001 (uncorrected, two-tailed).

**Table 3:** The correlations between short-term memory and language measures.

Variable	STM Forward	STM Backward	Melodic Perception	Pleasant-Sounding	Perceived Memorability	Difficulty Level	Familiar	Language Perception Aptitude
Intelligibility	.515 **	.343 **	.465 **	.039	.279 *	.319 **	.090	.397 **

* *p* < .05 (uncorrected, two-tailed). ** *p* < .001 (uncorrected, two-tailed).

**Table 4:** The multiple regression models explain the variance in the intelligibility total score.

Predictor	Partial Correlation (pr)	*p*-Value	Unstandardized B	Standardized Beta	95% Confidence Interval for B (Lower-/Upper Bound)	
Step 1: R = 0.49, F(1, 74) = 23.76, *p* < 0.001						
STMF	0.49	<.001	0.28	0.49	0.17	0.40
Step 2: R = 0.62, F(1, 73) = 16.43, *p* < 0.001						
STMF	0.44	<.001	0.94	1.06	0.00	0.00
Melodic perception	0.43	<.001	0.94	1.06	0.00	0.00
Step 3: R = 0.67, F(1, 72) = 8.82, *p* = 0.004						
STMF	0.41	<.001	0.20	0.35	0.10	0.31
Melodic perception	0.43	<.001	0.28	0.36	0.14	0.41
Melodic singing aptitude	0.33	.004	0.16	0.27	0.05	0.26
Step 4: R = 0.71, F(1, 71) = 6.63, *p* = 0.012						
STMF	0.37	.001	0.17	0.30	0.07	0.28
Melodic perception	0.42	<.001	0.26	0.34	0.13	0.39
Melodic singing aptitude	0.31	.007	0.14	0.24	0.04	0.24
Language perception aptitude	0.29	.012	1.6	0.23	0.36	2.83
Step 5: R = 0.73, F(1, 70) = 6.54, *p* = 0.013						
STMF	0.42	<.001	0.20	0.34	0.10	0.30
Melodic perception	0.30	.009	0.19	0.25	0.05	0.33
Melodic singing aptitude	0.33	.005	0.14	0.24	0.04	0.24
Language perception aptitude	0.30	.009	1.60	0.23	0.40	2.79
Perceived memorability	0.29	.013	0.16	0.23	0.04	0.29
Dependent variable: Intelligibility total						

**Table 5:** The correlational analysis of the behavioral perceptual language ratings and the selected music variables.

Variable	AMMA Tonal Aptitude	AMMA Rhythmic Aptitude	Melodic Singing Aptitude	Rhythmic Singing Aptitude
Melodic perception	.285 *	.339 **	.123	.138
Perceived memorability	.212	.251 *	.011	.025
Difficulty level	.137	.080	.384 **	.380 **

* *p* < .05 (uncorrected, two-tailed). ** *p* < .001 (uncorrected, two-tailed).

## Data Availability

The data are contained in the article or Supplementary Materials.

## Supplementary Materials

The following supporting information can be downloaded at: https://www.mdpi.com/article/10.3390/jintelligence11030043/s1, Tables S1–S7: Intraclass correlation coefficients of the intelligibility ratings of the in the six languages and the intraclass correlation coefficients for the melodic and rhythmic singing ratings; Figure S1: Singing task; Table S8: Correlations between familiarity musical variables; Table S9: Correlations between pleasant-sounding and musical variables.
