# Supplementary material for: What Makes a Foreign Language Intelligible? An Examination of the Impact of Musical Ability and Individual Differences on Language Perception and How Intelligible Foreign Languages Appear
Source: J Intell. 2023 Feb 23;11(3):43. doi: 10.3390/jintelligence11030043 (PMC10057304; doi:10.3390/jintelligence11030043)
Supplement: Supplementary file 1 [file jintelligence-11-00043-s001.zip › jintelligence-2136601-supplementary.pdf]

Intraclass coefficients

The tables S1 – S6 below show the intraclass correlation coefficients of the intelligibility ratings of the six languages and table S7 the intraclass correlation coefficients for the melodic and rhythmic singing ratings. We used a two- way mixed effects models where people effects were random and measures effects were fixed. For the language performances we had 3-6 raters for the tasks. Two language tasks consisted of nine and two language tasks of eleven syllables in each of the six languages. Sentence 1 and 2 represent the nine syllable samples for the respective languages, while sentence 3 and 4 illustrate the eleven syllable long sentence material. The singing criteria were rated by four singing teachers who evaluated the singing performance for how well the participants sustained the melody and rhythmical structure of the parts of the songs. Singing 1 and 2 represent the ratings for the melody, while singing 3 and 4 illustrates the ratings on the rhythmic criteria (see tables S7). The results of the intraclass correlation coefficients have shown that the ratings were reliable were at least of the accepted statistical value of 0.7 or above.

Table S1 illustrates the intraclass correlation coefficient of the Russian language ratings.

RUSSIAN	Number of Raters	Intraclass Correlation	*F Test with True Value 0*			
			Value	df1	df2	Sig.
Average measures of sentence 1	3	.796	7.002	79	158	.000
Average measures of sentence 2	3	.816	6.940	79	158	.000
Average measures of sentence 3	3	.779	6.327	79	158	.000
Average measures of sentence 4	3	.814	6.447	79	158	.000

Sentence 1 and 2: nine syllables; sentence 3 and 4: eleven syllables.

Table S2 illustrates the intraclass correlation coefficient of the Japanese language ratings.

JAPANESE	Number of Raters	Intraclass Correlation	*F Test with True Value 0*			
			Value	df1	df2	Sig.
Average measures of sentence 1	6	.913	15.739	79	395	.000
Average measures of sentence 2	6	.917	20.384	79	395	.000
Average measures of sentence 3	6	.870	13.332	79	395	.000
Average measures of sentence 4	6	.885	14.808	79	395	.000

Sentence 1 and 2: nine syllables; sentence 3 and 4: eleven syllables.

Table S3 illustrates the intraclass correlation coefficient of the Tagalog language ratings.

TAGALOG	Number of Raters	Intraclass Correlation	F Test with True Value 0			
			Value	df1	df2	Sig
Average measures of sentence 1	4	.742	4.669	79	237	.000
Average measures of sentence 2	4	.893	12.072	79	237	.000
Average measures of sentence 3	4	.845	7.171	79	237	.000
Average measures of sentence 4	4	.928	14.471	79	237	.000

Sentence 1 and 2: nine syllables; sentence 3 and 4: eleven syllables.

Table S4 illustrates the intraclass correlation coefficient of the Mandarin language ratings.

MANDARIN	Number of Raters	Intraclass Correlation	F Test with True Value 0			
			Value	df1	df2	Sig
Average measures of sentence 1	5	.754	7.436	79	316	.000
Average measures of sentence 2	5	.699	5.833	79	316	.000
Average measures of sentence 3	5	.723	5.050	79	316	.000
Average measures of sentence 4	5	.734	5.854	79	316	.000

Sentence 1 and 2: nine syllables; sentence 3 and 4: eleven syllables.

Table S5 illustrates the intraclass correlation coefficient of the Farsi language ratings.

FARSI	Number of Raters	Intraclass Correlation	F Test with True Value 0			
			Value	df1	df2	Sig
Average measures of sentence 1	4	.936	20.764	79	237	.000
Average measures of sentence 2	4	.916	16.818	79	237	.000
Average measures of sentence 3	4	.848	8.956	79	237	.000
Average measures of sentence 4	4	.889	13,507	79	237	.000

Sentence 1 and 2: nine syllables; sentence 3 and 4: eleven syllables.

Table S6 illustrates the intraclass correlation coefficient of the Thai language ratings.

THAI	Number of Raters	Intraclass Correlation	F Test with True Value 0

			Value	df1	df2	Sig
Average measures of sentence 1	5	.719	7.144	79	316	.000
Average measures of sentence 2	5	.763	5.696	79	316	.000
Average measures of sentence 3	5	.727	4.798	79	316	.000
Average measures of sentence 4	5	.760	5.353	79	316	.000

Sentence 1 and 2: nine syllables; sentence 3 and 4: eleven syllables.

Table S7 illustrates the intraclass correlation coefficient of the singing ratings.

SINGING	Number of Raters	Intraclass Correlation	*F Test with True Value 0*			
			Value	df1	df2	Sig
Average measures of singing 1 melody	4	.862	9.068	79	237	.000
Average measures of singing 2 melody	4	.935	15.475	79	237	.000
Average measures of singing 3 rhythm	4	.782	6.087	79	237	.000
Average measures of singing 4 rhythm	4	.886	8.999	79	237	.000

Singing 1 melody represents intraclass correlation coefficient of the short part of the melodic ratings, while singing 2 melody shows the intraclass correlation coefficient of the longer part of the melodic ratings. Singing 3 rhythm represents the intraclass correlation coefficient of the short part of the rhythmic ratings, while singing 4 rhythm shows the intraclass correlation coefficient of the longer part of the rhythmic ratings.

Figure S1

The figure below shows the singing tasks of the unfamiliar song. For the first part the participants were introduced to sing "whenever I miss, whenever I miss, I miss your smiling". The second part of the song was a further extension of the first part and the lyrics were as follows: "whenever I miss, whenever I miss, I miss your smiling, whenever I try, I try to fake a little smile".

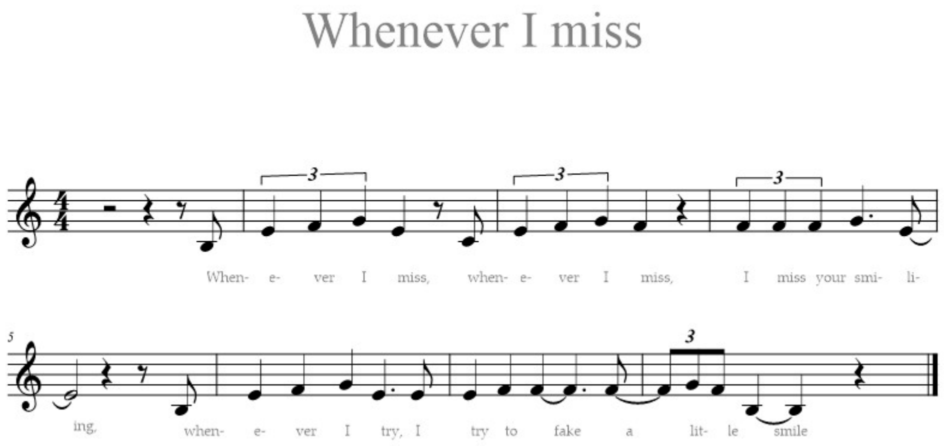

Figure S1. Short sequences of the unfamiliar song.

Correlational analyses

Correlational analyses were applied in order to provide information about the relationship between the variables of interest. The tables S8 and S9 below show the correlations of the familiarity and the pleasant-sounding score and the musical variables under consideration. As outlined, we did not find any correlation between the musical variables familiarity and pleasant-sounding.

Table S8. Correlations familiarity.

Variable	AMMA tonal aptitude	AMMA rhythmic aptitude	Melodic singing aptitude	Rhythmic singing aptitude
Familiarity	.008	-.007	.192	.198

*p < .05 (uncorrected, two-tailed). ** p < .001 (uncorrected, two-tailed).

Table S9. Correlations pleasant sounding.

Variable	AMMA tonal aptitude	AMMA rhythmic aptitude	Melodic singing aptitude	Rhythmic singing aptitude
Pleasant sounding	.118	.153	-.057	-.074

*p < .05 (uncorrected, two-tailed). ** p < .001 (uncorrected, two-tailed).
